# Strong Expansion of Human Regulatory T Cells for Adoptive Cell Therapy Results in Epigenetic Changes Which May Impact Their Survival and Function

**DOI:** 10.3389/fcell.2021.751590

**Published:** 2021-11-18

**Authors:** Kristy Ou, Dania Hamo, Anne Schulze, Andy Roemhild, Daniel Kaiser, Gilles Gasparoni, Abdulrahman Salhab, Ghazaleh Zarrinrad, Leila Amini, Stephan Schlickeiser, Mathias Streitz, Jörn Walter, Hans-Dieter Volk, Michael Schmueck-Henneresse, Petra Reinke, Julia K. Polansky

**Affiliations:** ^1^BIH Center for Regenerative Therapies (BCRT), Berlin Institute of Health at Charité - Universitätsmedizin Berlin, Berlin, Germany; ^2^Berlin Center for Advanced Therapies (BeCAT), Charité - Universitätsmedizin Berlin, Berlin, Germany; ^3^Department of Genetics and Epigenetics, Saarland University, Saarbrücken, Germany; ^4^Institute of Medical Immunology, Charité - Universitätsmedizin Berlin, Berlin, Germany; ^5^German Rheumatism Research Centre (DRFZ) Berlin, Berlin, Germany

**Keywords:** regulatory T cells, advanced therapy medicinal products, DNA methylation, biomarker, adoptive cell therapy, good manufacturing practice

## Abstract

Adoptive transfer of regulatory T cells (Treg) is a promising new therapeutic option to treat detrimental inflammatory conditions after transplantation and during autoimmune disease. To reach sufficient cell yield for treatment, *ex vivo* isolated autologous or allogenic Tregs need to be expanded extensively *in vitro* during manufacturing of the Treg product. However, repetitive cycles of restimulation and prolonged culture have been shown to impact T cell phenotypes, functionality and fitness. It is therefore critical to scrutinize the molecular changes which occur during T cell product generation, and reexamine current manufacturing practices. We performed genome-wide DNA methylation profiling of cells throughout the manufacturing process of a polyclonal Treg product that has proven safety and hints of therapeutic efficacy in kidney transplant patients. We found progressive DNA methylation changes over the duration of culture, which were donor-independent and reproducible between manufacturing runs. Differentially methylated regions (DMRs) in the final products were significantly enriched at promoters and enhancers of genes implicated in T cell activation. Additionally, significant hypomethylation did also occur in promoters of genes implicated in functional exhaustion in conventional T cells, some of which, however, have been reported to strengthen immunosuppressive effector function in Tregs. At the same time, a set of reported Treg-specific demethylated regions increased methylation levels with culture, indicating a possible destabilization of Treg identity during manufacturing, which was independent of the purity of the starting material. Together, our results indicate that the repetitive TCR-mediated stimulation lead to epigenetic changes that might impact functionality of Treg products in multiple ways, by possibly shifting to an effector Treg phenotype with enhanced functional activity or by risking destabilization of Treg identity and impaired TCR activation. Our analyses also illustrate the value of epigenetic profiling for the evaluation of T cell product manufacturing pipelines, which might open new avenues for the improvement of current adoptive Treg therapies with relevance for conventional effector T cell products.

## Introduction

T Lymphocytes are one of the most promising effectors for adoptive cellular therapy (ACT) by facilitating target-specific immune interventions, as has recently been impressively demonstrated in clinical practice (Guedan et al., 2019). ACTs comprised of regulatory T cells (Tregs) are currently under extensive clinical testing (Sharabi et al., 2018) to prevent undesired inflammation and immune reactivity during autoimmunity (Bluestone et al., 2015; Elias and Rudensky, 2019; Ryba-Stanislawowska et al., 2019), after solid organ transplantation (Romano et al., 2019; Sawitzki et al., 2020) or during graft-versus-host-disease (GvHD) after hematopoietic stem cell transplantation (Bertaina and Roncarolo, 2019; Elias and Rudensky, 2019; Mancusi et al., 2019). Despite previous concerns of inducing systemic over-suppression and increased risk for tumor formation and infections, studies from different centers including our own have shown a very good safety profile and even hints of efficacy: A recent phase 1/2a clinical trial (The ONE study; Roemhild et al., 2020; Sawitzki et al., 2020) testing Treg products from our facility demonstrated that drug-mediated immunosuppression could successfully be sustainably reduced in most kidney transplant recipients (Roemhild et al., 2020). Still, reduction of immunosuppression was not tolerated by a few patients and complete weaning was not feasible at all, indicating that there is room for improvement in the fitness, function, or engraftment of the Treg products. In addition, other critical challenges remain such as the standardization of manufacturing procedures and of quality control parameters, as well as the high costs of production (Trzonkowski et al., 2015; Raffin et al., 2020).

For Treg products, functional quality assessment remains a major challenge (Trzonkowski et al., 2015; Fuchs et al., 2017), as the only widely used *in vitro* suppression assay (Brusko et al., 2007; Venken et al., 2007) poorly represents the *in vivo* functionality of Tregs (Scheffold et al., 2005; Collison and Vignali, 2011; Wendering et al., 2019). In addition, classical Treg biomarkers (e.g., FOXP3, CD25, and lack of CD127 expression) can also be found on contaminating activated pro-inflammatory conventional T cells (Tran et al., 2007; Wang et al., 2007; Kmieciak et al., 2009). Furthermore, the quality of the final Treg ACT product is highly dependent on the donor and on the purity of the starting material. Until recently, highly pure Treg starting populations from peripheral blood could not be achieved due to the lack of appropriate flow-cytometric sorting devices which would be accepted by most European regulatory authorities. In addition, extensive TCR stimulation-driven *in vitro* expansion of the starting material is required, which poses risks for the outgrowth of contaminating pro-inflammatory T cells (Battaglia et al., 2005, 2006), possible loss of Treg identity (Marek et al., 2011; Bailey-Bucktrout et al., 2013), terminal differentiation, and perhaps functional exhaustion or senescence as reported for conventional T cells (Chou and Effros, 2013; Wherry and Kurachi, 2015; Okuda et al., 2019). Many different protocols for Treg product manufacturing have been developed and tested (Duggleby et al., 2018; Fraser et al., 2018; MacDonald et al., 2019; Alzhrani et al., 2020), but are difficult to compare due to the lack of a standardized quality control procedure (Fuchs et al., 2017). It is therefore of great importance to assess functional or molecular alterations which occur during Treg product generation in order to identify current therapeutic limitations and develop strategies for improvement.

To address this, we characterized manufacturing-induced epigenetic changes at the DNA methylation level during the generation of Treg products at our center. Epigenetic mechanisms define the potential for gene expression at individual loci through chromatin permissibility, and can give insight into both current and future functional states (Youngblood et al., 2013; Durek et al., 2016; Abdelsamed et al., 2017; Delacher et al., 2017, 2021). For T cells in particular, the functional state can be shaped by epigenetic remodeling in response to extracellular signaling cues as was recently shown for exhausted T cells (Pauken et al., 2016; Ghoneim et al., 2017). Therefore, analyzing manufacturing-induced epigenetic changes may be informative for predicting the cellular state and function of the final Treg product. In our analyses, we determined that the manufacturing process introduces wide-spread, reproducible, and progressive changes in the DNA methylome of the Treg products, including at loci important for T cell activation and Treg identity.

## Materials and Methods

### Ethics

All donors donating blood for this study provided written informed consent for their participation. Study procedures were approved by the Ethics Committee of the Charité - Universitätsmedizin Berlin.

### Manufacturing of 1st Generation Products

Peripheral blood from healthy donors was used for enrichment of natural regulatory T cells (nTreg). Two consecutive CliniMACS runs, compromising a CD8^+^ cell depletion followed by a CD25^+^ cell selection, were performed according to the manufacturer‘s instructions (Miltenyi Biotec). Target cell fraction was cultured in complete nTreg-medium, containing X-Vivo 15 medium (Lonza) supplemented with fetal calf serum (FCS, Hyclone), recombinant human IL-2 (Miltenyi Biotec) and Rapamycin (Pfizer). Depending on cell numbers, expansion process of Tregs was performed in 96-well plates and 24-well plates, respectively, for 23 days, using complete nTreg-medium and repetitive stimulation with anti-CD3/CD28 MACSiBead particles (Treg Activation/Expansion Kit, Miltenyi Biotec). Cells were collected for downstream DNA methylation analysis at several time-points during culture.

### Manufacturing of 2nd Generation Products

Peripheral blood was obtained from healthy donors, and peripheral blood mononuclear cells (PBMCs) were isolated by gradient centrifugation with Biocoll (Biochrom). The CD4^+^ CD25^high^ CD127^–^ Treg cells were sorted by a flow cytometry-based sorting system, the MACSQuant Tyto Cell Sorter (Miltenyi Biotec). Staining was performed using fluorophore-conjugated human anti-CD4, anti-CD25 and anti-CD127 antibodies (all Miltenyi Biotec), achieving purities of over 90%. Treg cells were cultured in a 96-well round-bottom plates (Eppendorf) with complete nTreg-medium compromising X-Vivo 15 medium (Lonza) supplemented with FCS (Biochrom), Penicillin/Streptomycin (Biochrom), Rapamycin (Sigma-Aldrich) and recombinant human IL-2 (Novartis). Treg cells were repetitively stimulated with anti-CD3/CD28 MACSiBead particles (Treg Activation/Expansion Kit, Miltenyi Biotec) for a total of 8 times throughout culture for 21 days.

### Isolation and Culture of FACS-Sorted Tregs and CD4^+^ Memory T Cells

PBMCs were isolated from 50 mL of peripheral blood obtained from healthy donors, by Ficoll-Paque Plus (Thermo Fisher Scientific) density gradient centrifugation followed by erythrocyte lysis using Buffer EL (Qiagen). After CD4 enrichment using magnetic cell separation with the autoMACS instrument (Miltenyi Biotec) using CD4 MicroBeads (Miltenyi Biotec), cells were left overnight at 4°C in basal cell culture medium [RPMI 1640 Medium with GlutaMAX Supplement-10 (Thermo Fisher Scientific), 10% FCS (Corning), 50 nM 2-mercaptoethanol (Thermo Fisher Scientific), 1 mM pyruvate (Biochrom), and 25 mM HEPES (Gibco)]. The following day, CD4^+^ memory T cells (CD4^+^ CD25^+^ CD127^+^ CD45RO^+^) and Tregs (CD4^+^ CD25^+^ CD127^–^) were FACS-sorted with a BD FACSAria II SORP (Becton Dickinson). Sorted CD4^+^ memory T cells and Tregs were initially cultured in 96-well round-bottom plates (Greiner Bio-One) with the aforementioned cell culture media supplemented with 500 IU/mL rhIL-2 (R&D Systems) and 10 nM Rapamycin (STEMCELL Technologies) (day 0). The following day, cells were stimulated with anti-CD3/CD28 MACSiBead particles (Treg Activation/Expansion Kit, Miltenyi Biotec) according to manufacturer’s guidelines. Cells were stimulated at the same time points as the 1st and 2nd generation products, and collected for downstream DNA methylation analysis.

### Surface Expression Profiling

GMP-compliant Treg products and PBMCs were stained using fluorescently conjugated monoclonal antibodies for CD3 (BV650, clone OKT3), CD4 (PerCP-Cy5.5, clone SK3), CD8 (BV510, clone RPA-T8), CD127 (APC-A700, clone A019D5), and CD25 (APC, clone M-A251) at 4°C for 30 min. Backbone-stained samples were subsequently split to be stained for further 30 min with PE-conjugated antibodies for different human surface antigens: CD5 (clone UCHT2), CD80 (clone 2D10), CD86 (clone IT2.2), CD59 [clone p282 (H19)], CD160 (clone BY55), CD279 (clone EH12.2H7), CD366 (clone F38-2E2), and TIGIT (clone A15153G). All antibodies were purchased from BioLegend. To exclude dead cells, LIVE/DEAD Fixable Blue Dead Cell Stain dye (Thermo Fisher Scientific) was added. Lymphocytes were gated on the basis of the forward scatter (FSC) vs. side scatter (SSC) profile after exclusion of doublets via FSC-Height vs. FSC-Area. Tregs were defined as CD25^high^ CD127^low^ CD4^+^ CD8^–^ CD3^+^ and conventional T cells (Tcon) as CD25^–^ CD127^+^CD4^+^ CD8^–^ CD3^+^. Cells were analyzed on a Cytoflex LX (Beckmann Coulter) flow cytometer. Cytometric raw data were analyzed using Kaluza^TM^ software (Beckman Coulter), mean expression values were visualized using the *nautilus* R package (available from^1^).

### Methylation Profiling

Genomic DNA of cell pellets was isolated using AllPrep DNA/RNA Mini Kit (Qiagen) or Zymo’s Quick-DNA MicroPREP Kit (Zymo Research), following manufacturer’s instructions. DNA concentration was assessed with Qubit dsDNA HS Assay Kit and the Qubit Fluorometer (Molecular Probes/Life Technologies). 200–250 ng of genomic DNA was used as input for bisulfite conversion, which was performed with Zymo’s EZ DNA Methylation-Gold Kit (Zymo Research) or Zymo’s EZ DNA Methylation Kit (Zymo Research). Methylation analysis was performed with the Infinium MethylationEPIC Kit (Illumina EPIC-8 BeadChip) following the manufacturer’s instructions. Bead Chips were imaged on Illumina’s Microarray Scanner iScan.

### Data Processing and Analysis

All analyses were conducted in the R statistical software (Version > 4.0).

#### Preprocessing Raw Data

Raw intensity data files (IDAT) from Bead Array scans were preprocessed using *minfi* version 1.36.0. Probes were removed if they did not meet the detection *p*-value threshold (*p* < 0.05), were found to be cross reactive (Pidsley et al., 2016), or changes in manufacturing process (Infinium MethylationEPIC v1.0 B5 Release Date). Raw data was normalized by quantile normalization, after which M values were extracted. Due to sample processing at different institutes, samples were subjected to batch correction using the *ComBat* function from *sva* version 3.38.0 (Leek et al., 2012). Lastly, CpGs within 3 nucleotides away from a SNP/variant was removed with the function *rmSNPandCH* from *DMRcate* version 2.4.1 (Peters et al., 2015).

#### Differential Methylation Analysis

CpGs on chromosomes X or Y were removed prior to differential methylation analysis. Significant (FDR < 0.05) differentially methylated positions (DMPs) were identified using the *cpg.annotate* function in *DMRcate*, which were then used to identify differentially methylated regions (DMRs) using the *dmrcate* function with lambda = 500 and C = 3. DMRs with a Stouffer’s score < 0.05 were kept for further analysis. CpGs and DMRs were annotated to genomic features and genes using *annotatePeak* from the *ChIPseeker* version 1.26.2 (Yu et al., 2015).

#### Publicly Available Data

##### Partially Methylated Domains

Bed files containing genomic annotations for PMDs in CD4 central memory (CM), effector memory (EM), effector memory RA (TEMRA), and Tregs were downloaded from Gene Expression Omnibus GSE113405 (Salhab et al., 2018). Due to the heterogeneous starting material of the first generation Tregs, an overlapping core set of PMDs was identified through *findOverlaps* function in *GenomicRanges*.

##### Whole Genome Bisulfite Sequencing

CpG methylation read counts from WGBS data of HIV-reactive CD8 T cells and CD8 effector memory were downloaded from Gene Expression Omnibus GSE144693 (Abdelsamed et al., 2020). Only CpGs that were covered at least 10X were considered for analysis. *dmrcate* was also used to identify DMRs between exhausted HIV-reactive CD8 T cells and CD8 effector memory cells using the parameters with lambda = 500 and C = 3.

##### ChromHMM Chromatin States

ChromHMM chromatin states of Treg and CD4 memory cells were downloaded from the NIH Roadmap Epigenomics Project portal https://egg2.wustl.edu/roadmap/web_portal/

#### Statistical Analysis and Data Visualization

Gene set enrichment analysis was performed with the package *fgsea* version 1.16.0 (Korotkevich et al., 2021). Gene ontology enrichment analysis was performed with *clusterProfiler* version 3.18.1 (Yu et al., 2012). For *T* test statistical analysis, the function *stat_compare_means* from *ggpubr* version 0.4.0 was used^2^. Heatmaps were generated using *ComplexHeatmap* version 2.6.2 (Gu et al., 2016). Alluvial plot was generated with *ggalluvial* version 0.12.3^3^. Density plot margins for PCA plot was generated with *ggExtra* version 0.9^4^. All other plots were generated with *ggplot2* version 3.3.5.

## Results

### *In vitro* Expansion During Treg Product Generation Induces Changes in the DNA Methylome

**FIGURE 1:**
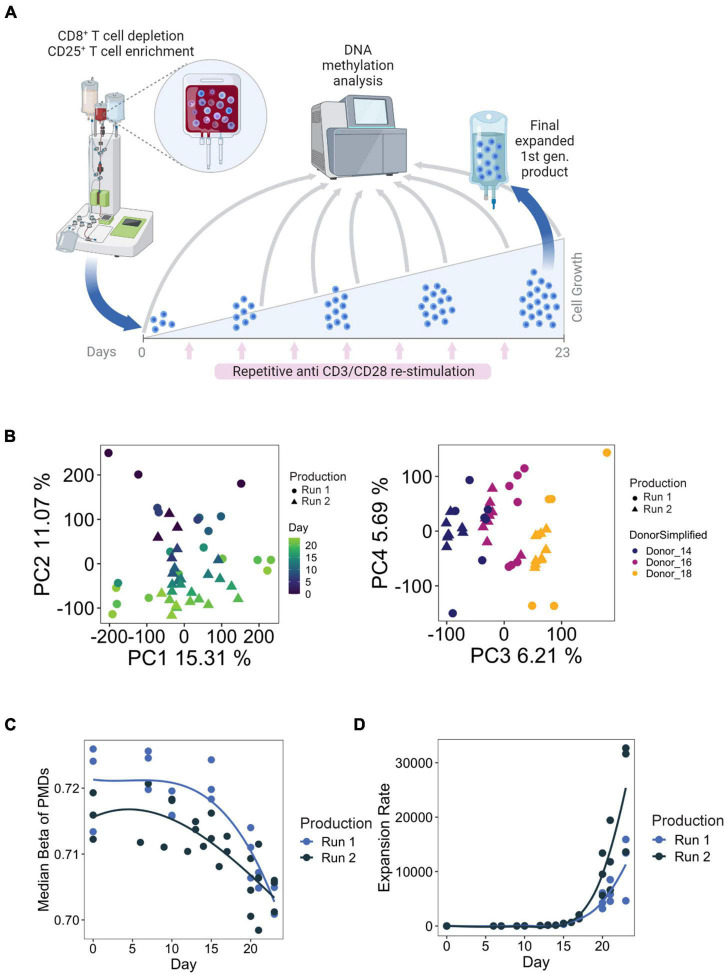
**(A)** Example manufacturing run for first-generation Treg products. Briefly, Tregs were enriched through CD8^+^ depletion followed by CD25^+^ enrichment using the CliniMACS^®^ system from whole blood. Tregs are cultured over the course of 23 days, with 8 total stimulations with anti-CD3/CD28 microbeads. Time points used for DNA methylation analysis are indicated with the gray arrows. Figure created with BioRender.com. **(B)** PCA analysis of samples taken from all available time points from both manufacturing runs. **(C,D)** Median methylation values of partially methylated domains (PMDs) (left) and cumulated expansion rate (right) during Treg product generation.

Treg products from three healthy donors were generated twice by two indepenent manufacturing runs. These first generation (1st gen.) Treg products are generated from peripheral blood-derived starting material which is depleted of CD8^+^ cells and enriched for CD25-expressing cells using the CliniMACS system, yielding a starting populations enriched for CD8^–^ CD4^+^ CD25^+^ Tregs. For generation of the final product, the cells undergo expansion culture for 23 days with repetitive TCR stimulation (using antiCD3/CD28 microbeads, 8 stimulations in total) in the presence of IL2 and Rapamycin. Supplementation of Rapamycin is meant to inhibit the outgrowth of contaminating conventional proinflammatory CD4^+^ T cells (Battaglia et al., 2005). We harvested samples at the starting (day 0), final (day 23), and relevant additional time points during cell expansion (Figure 1A). The samples were subjected to genome-wide methylation analysis using the Illumina Methylation EPIC microarray, which covers a selection of 850,000 CpG methylation sites genome-wide with a special emphasis on regulatory elements such as promoters and enhancers.

Principal component analysis (PCA) of the EPIC array methylation profiles demonstrated that the number of days in culture had a substantial impact on the DNA methylome, as this feature accounted for PC2 (11% of the total variation in the data set) (Figure 1B). Inter-donor variances could also be observed and were reflected on PC3 (Figure 1B). Importantly, within the same donor, the samples did not separate based on production runs. These observations indicate that there is a progressive change in DNA methylation with prolonged culture, which is reproducible between donors and production runs. We also explored methylation changes at partially methylated domains (PMDs) of the genome, which are large transcriptionally silenced regions characterized by a highly disorganized methylation pattern, a generally reduced DNA methylation level and poor gene content that undergo methylation loss as a result of strong episodes of proliferation (Salhab et al., 2018; Decato et al., 2020). We indeed observed a loss of PMD methylation with increased expansion rate (Figure 1C), which is consistent with previous findings that demonstrated that accumulation of cell divisions drive PMD hypomethylation in cancer and aging (Zhou et al., 2018), and during T cell differentiation (Durek et al., 2016).

We next performed differential methylation analysis to identify genetic loci undergoing significant methylation changes during Treg product generation. Increased DNA methylation in promoters, first introns, and enhancers generally correlate with transcriptional downregulation (Varley et al., 2013; Anastasiadi et al., 2018), while hypomethylation correlates with transcriptional activation. In contrast, hypermethylation at gene bodies (mainly on exons) has been reported to correlate with increased transcription (Ball et al., 2009) and increased methylation at alternatively spliced exons has been implicated in differential splicing by mediating exon retention during transcription (Sun et al., 2020).

**FIGURE 2:**
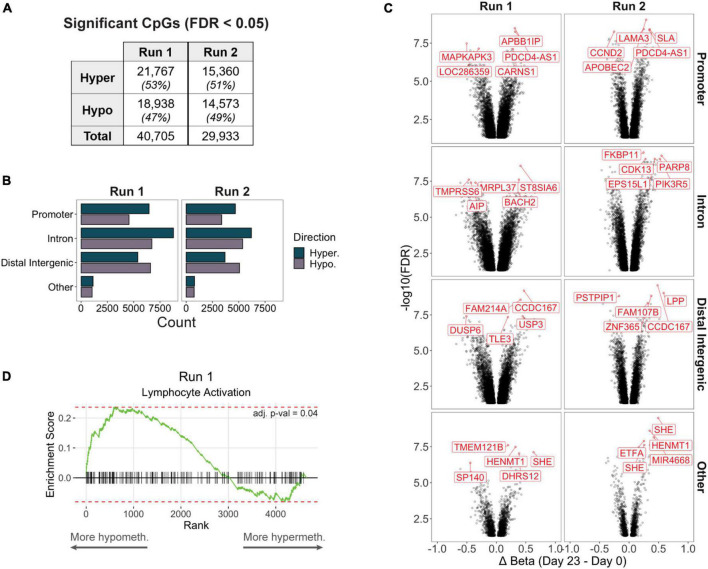
**(A)** Significantly differentially methylated CpGs (differentially methylated positions, DMPs) between day 0 and day 23 products in each production run. **(B)** Counts of hyper- or hypomethylated DMPs mapping to the promoter, intron, distal intergenic, or other (5′ or 3′ UTRs, exons, or immediate downstream) region of a gene. **(C)** Volcano plots of significant DMPs (FDR < 0.05) stratified by genic annotation. The top 5 most significant DMPs (stratified by genic annotation) are highlighted. **(D)** In Run 1, genes that contained at least 1 promoter DMP but did not contain any differentially methylated regions (DMRs, including at least 3 CpGs) were significantly enriched for the Lymphocyte Activation pathway (adj. *p*-val < 0.05).

Although we focus much of our later analyses on differentially methylated regions (DMRs) containing at least 3 CpGs, we also characterized differentially methylated positions (DMPs) of individual CpGs in case biologically important loci were not sufficiently covered by multiple adjacent CpG probes due to microarray design limitations. We detected approximately 40,700 and 30,000 CpGs that were differentially methylated in production Runs 1 and 2, respectively, between the final (day 23) and starting (day 0) products (Figure 2A, Supplementary Table 1). For both runs, majority of DMPs mapped to introns (38%), followed by distal intergenic regions (29%) and promoters (27%) (Figure 2B). Other DMPs mapping to the 5′ or 3′ UTRs, exons, and genomic regions immediately downstream of the gene body (<300 bp) comprised only 5% of all significant sites. Furthermore, promoters and introns consistently had a higher number of hypermethylated DMPs, whereas hypomethylated DMPs were more frequent in intergenic regions. Interestingly, a few CpGs were consistently among the top 5 most significant DMPs (stratified by genic annotation) in both production runs, such as a hypermethylated DMP mapping to the promoter of *PDCD4-AS1*, a long non-coding RNA that has been shown to positively regulate *PDCD4*, a tumor suppressor gene that has also been implicated in autoimmune disorders (Hilliard et al., 2006; Jadaliha et al., 2018; Figure 2C).

We then performed DMR analysis to identify genomic regions containing adjacent differentially methylated DMPs, and identified 1,753 DMRs associated with 1,515 unique genes in Run 1, and 1,246 DMRs associated with 1,103 genes in Run 2; the remaining 10,852 genes from Run 1 and 9,597 genes from Run 2 did not contain a DMR despite having at least 1 significantly DMP. However, these non-DMR DMPs may still function as important regulators of Treg or T cell biological processes: When we performed gene set enrichment analysis of Run 1 promoter DMP genes that were not represented by any DMRs, we found significant enrichment for genes important for lymphocyte activation (Figure 2D).

### Consistent Epigenetic Remodeling Occurs at Genes Important for T Cell Activation and Differentiation

**FIGURE 3:**
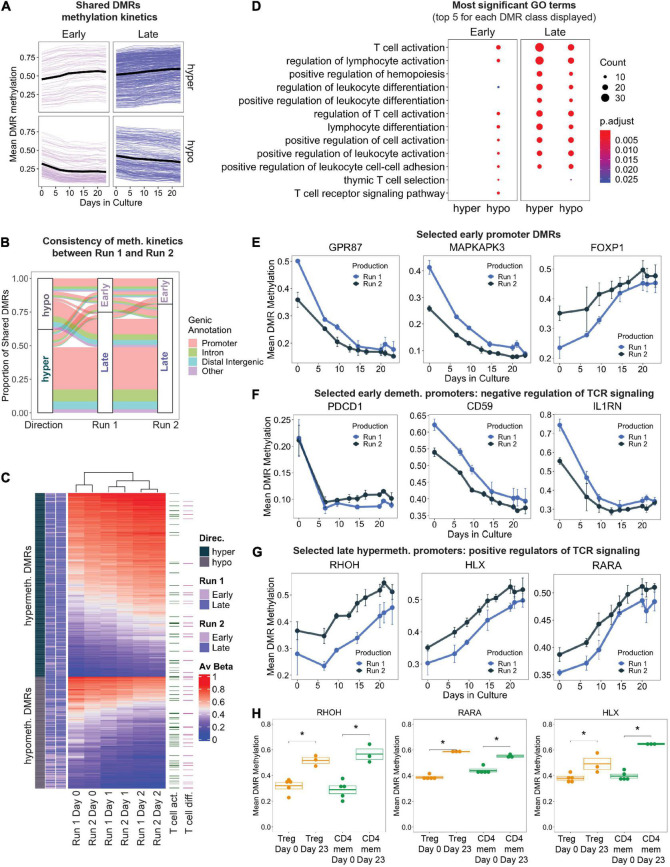
**(A)** Change in mean DMR methylation of shared DMRs over the 23 days of *in vitro* expansion. Early DMRs were defined as being significantly differentially methylated in day 10 – day 0 (and remained significant in day 23 – day 0), and Late DMRs were only significantly differentially methylated at day 23 – day 0. Each line displays one DMR, bold black line displays the mean. **(B)** Display of all shared DMRs according to their directionality of methylation change (hyper/hypo) and to their Early/Late classification (as in **A**) in production Run1 and 2. Color code indicates genic annotation of the DMR. **(C)** Unsupervised hierarchical clustering of shared DMRs in both production runs, with DMRs mapping to genes implicated in T cell activation and T cell differentiation (GO terms 0042110 and 0030217) highlighted. **(D)** Gene ontology enrichment analysis of shared DMRs that were consistently “Early” or “Late” between both production runs, stratified by directionality. The top 5 GO terms (ranked by adjusted *p*-value) for each DMR class are represented, many of which overlap. Size of dots indicates number of genes associated with GO term, and color indicates adjusted *p*-value. **(E–G)** Level of DNA methylation on selected DMRs in known T cell genes. **(H)** DNA methylation at the promoters of *RHOH*, *HLX*, and *RARA* in FACS-sorted Tregs and memory CD4^+^ T cells (CD4mem) before (Day 0) and after frequent CD3/CD28 stimulation (Day 23). ^∗^denotes *p*.val < 0.05 (*T* test).

We next focused on significant DMRs that were present in both production runs as they would reflect reproducible methylation changes during Treg product generation. We defined “shared DMRs” as production run 1 DMRs overlapping with run 2 DMRs by 50% or more. Furthermore, we were also interested in delineating early epigenetic changes in response to initial activation versus changes occurring only later, the latter resulting from progressive changes due to prolonged culture. We therefore labeled shared DMRs as “Early” (first identified in day 10 vs. day 0 comparison and still significant in day 23 *versus* day 0) or “Late” (first identified in day 23 vs. day 0 in both production runs). As expected, Early DMRs displayed stronger initial methylation change within the first 10 days of culture (indicated by the bend in the methylation time series traces) (Figure 3A).

In total, there were 838 shared DMRs (Supplementary Table 2), with 62% hypermethylated by the final time point (Figure 3B). Although the directionality (hyper- vs. hypomethylated) of the DMRs was consistent between the two production runs, the methylation kinetics (“Early” or “Late”) differed slightly (Figure 3B): 16% of shared DMRs had different kinetics between the two runs. This may be a result of variability in expansion rates between the two runs (Figure 1D), differences in the proportion of contaminating cell types in the starting day 0 cultures, or simply lack of statistical power. Gene promoters accounted for the majority of all shared DMRs (63%), followed by introns and distal intergenic elements, both of which frequently contain enhancers.

Unsupervised hierarchical clustering based on shared DMR mean methylation values at days 0, 10, and 23 depicted samples of the same time points clustering together regardless of their corresponding production run (Figure 3C). Genes implicated in T cell activation and differentiation were present in both hypo- and hypermethylated DMRs. We performed Gene Ontology (GO) enrichment analysis on the shared DMRs that were stratified by directionality and methylation kinetics to evaluate biological processes that may be regulated by epigenetic mechanisms. Genes that were significantly hypomethylated early during manufacturing are implicated in T cell activation, differentiation, adhesion, and other related pathways (Figure 3D). Enrichment of these GO terms was maintained even in the late hypomethylated DMRs. Examples of genes undergoing early strong promoter hypomethylation (>15%) include genes important for cell signaling, intracellular signal transduction, and proliferation such as *GPR87* and *MAPKAPK3* (Figure 3E; Zhang et al., 2010; Cargnello and Roux, 2011). Genes containing early hypermethylated DMRs did not enrich for any GO term, but still contained interesting candidate genes. We observed strong hypermethylation at the promoter *FOXP1* (Figure 3E), perhaps indicating a path toward terminal differentiation of the Treg products as DNA methylation-controlled transcriptional downregulation of *FOXP1* has been shown to correlate with end-differentiated T cells (Goronzy and Weyand, 2012; Durek et al., 2016).

We also observed early DNA hypomethylation of genes reported to negatively modulate TCR-mediated signaling, such as *PDCD1*, *CD59* (Sivasankar et al., 2009), and *IL1RN* (McIntyre et al., 1991; Arend et al., 1998; Figure 3F). This may indicate the beginning of a negative feedback loop that is normally part of a physiological response to restrict T cell effector function in order to prevent overshooting of the immune response during antigen encounter. Furthermore, many DMRs mapping to genes important for T cell activation were significantly hypermethylated later in culture (Figure 3D). For example, a progressive increase in promoter methylation was detected at genes which positively regulate TCR signaling and T cell differentiation, such as *RHOH* (Wang et al., 2011), *HLX* (Allen et al., 1995), and *RARA* (Hall et al., 2011), suggesting transcriptional inactivation that may occur through repetitive stimulation-induced epigenetic reprogramming (Figure 3G). To demonstrate that hypermethylation of these TCR signaling loci occurs independently of starting material purity, we also profiled these promoters’ methylation status in FACS sorted primary conventional CD4^+^ memory T cells (CD4mem) and Tregs which have also been stimulated repeatedly with antiCD3/CD28 beads in similar culture conditions. Indeed, both Tregs and CD4mem at day 23 displayed significantly increased promoter methylation compared to the starting populations (day 0) (Figure 3H), indicating that frequent TCR stimulation for 23 days is capable of epigenetically reprogramming and potentially stably downregulating these critical genes.

### Genes Upregulated in Chronically Activated T Cells Undergo Significant Methylation and Surface Expression Changes in First Generation Treg Products

**FIGURE 4:**
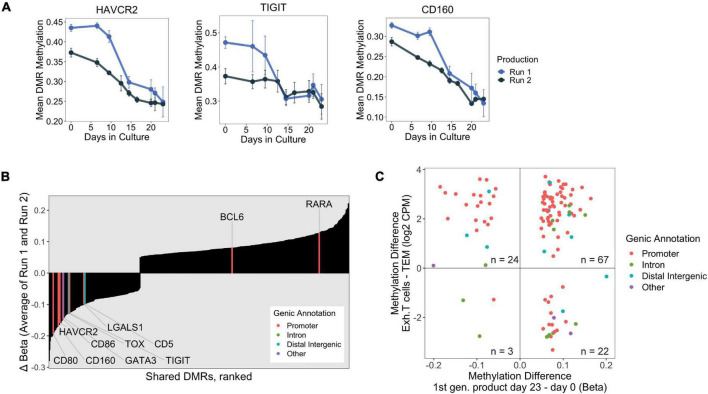
**(A)** DNA methylation changes over 23 days at the promoters of select exhaustion-associated loci. **(B)** Consistently “Late” shared DMRs were ranked by final change in methylation (Day 23 – Day 0, averaged over both production runs). Genes implicated in exhaustion, taken from publicly available datasets, are highlighted with associated genic annotation. Notably, many of the exhaustion associated genes are among the strongest hypomethylated DMRs. **(C)** Scatter plot displaying shared DMRs which overlapped with ExhDMRs identified from WGBS comparing exhausted CD8 T cells vs. CD8 effector memory T cells. The x-axis depicts methylation difference between day 23 and day 0 products (average of both runs), and y-axis depicts methylation difference between functionally exhausted CD8 T cells (Exh. T cells) and CD8 effector memory T cells (TEM).

We also identified promoter-associated Late DMRs that were significantly and strongly hypomethylated (methylation loss > 15%) at *HAVCR2* (encoding for TIM-3), *TIGIT*, and *CD160* (Figure 4A), which are co-inhibitory surface molecules that are upregulated in T cells and Tregs during prolonged antigen encounter. Although upregulation of these co-inhibitory surface molecules is often associated with exhaustion, a hypo-responsive and dysfunctional state in conventional T cells (Tcon) caused by persistent TCR signaling (reviewed in Blank et al., 2019), they are also linked to enhanced immunosuppressive capacity in Tregs (Sakuishi et al., 2013; Fuhrman et al., 2015; Liu et al., 2018).

We were interested in profiling methylation changes at additional genes which have been reported to be transcriptionally upregulated during prolonged antigen exposure. We curated a list of genes by combining T cell dysfunction/exhaustion gene modules from Singer et al. (2016) with genes identified in CD4^+^ exhausted T cells from Crawford et al. (2014), and checked whether these genes were present in our shared DMR dataset. Indeed, we found several genes known to be upregulated in exhausted T cells which were consistently differentially methylated during Treg product generation, with most of them experiencing strong hypomethylation at their promoters (Figure 4B). We confirmed that strong promoter hypomethylation corresponded to upregulation of these chronic activation-induced genes (i.e., HAVCR2/TIM-3, TIGIT, CD160, CD59, CD80, CD86, and CD5), as the final product had higher surface protein expression than either of the *ex vivo* Treg and Tcon populations present in the day 0 starting material (Supplementary Figure 1).

Epigenetic changes have also been correlated to the establishment of functional exhaustion, most well studied in the context of tumors and chronic infections (Khan et al., 2019; Scott et al., 2019). In a recent study conducted by Abdelsamed et al. (2020), the authors generated whole genome bisulfite sequencing (WGBS) data from CD8^+^ effector memory T cells (TEM) derived from healthy donors and patient-derived exhausted PD-1^+^ HIV-reactive CD8^+^ T cells (Texh). We took the opportunity to reanalyze this same WGBS dataset to define DNA methylation features distinguishing chronically activated CD8^+^ T cells (Texh) from TEM, and determined whether these epigenetic differences were also shared with our first generation products which have undergone repetitive TCR stimulation. Our analysis identified approximately 52,000 genomic regions that were differentially methylated between TEM and exhausted T cells, which we refer to as exhDMRs.

We rationalized that the consistently Late shared DMRs identified in our Treg products would likely contain exhaustion-associated changes because these late changes were likely induced by long-term TCR stimulation. We found that approximately 20% of all Late shared DMRs overlapped with the exhDMRs (116 overlapping DMRs of 585 Late shared DMRs, Supplementary Table 3), which is likely an underestimation due to the limited number of CpGs captured by the EPIC microarray. Of the overlapping DMRs, 91 were significantly hypermethylated in exhausted T cells compared to TEM, of which 74% were consistently hypermethylated in the final Treg products (day 23) compared to day 0 (Figure 4C). A vast majority (85%) of these hypermethylated DMRs also mapped to promoters of genes (highlighted red in Figure 4C), suggesting that a set of hypermethylated exhaustion-associated genes are similarly transcriptionally silenced by epigenetic mechanisms during *in vitro* manufacturing of Treg products.

### Treg-Specific Demethylated Loci Are Progressively Hypermethylated With Culture

Preservation of Treg immunosuppressive function and identity is a primary concern for the development of adoptive Treg therapies, as persistent TCR-mediated activation has been shown to correlate with downregulation of the lineage-defining transcription factor FOXP3, although perhaps only selectively in memory (CD45RA^–^) Tregs (Hoffmann et al., 2009; Arroyo Hornero et al., 2017; Guo et al., 2019). The downregulation of FOXP3 is linked to the hypermethylation of an intronic enhancer in the FOXP3 gene, the so-called FOXP3 Treg-Specific Demethylated Region (FOXP3-TSDR), which is used as an epigenetic marker to distinguish *de facto* Tregs from closely related conventional CD4^+^ memory T cells (CD4mem) due to its highly selective demethylated status only in Tregs (Huehn et al., 2009). The consequential gain of FOXP3-TSDR methylation in response to persistent TCR stimulation demonstrates the need to profile additional culture-induced epigenetic changes at other Treg-specific loci, as these changes may be predictive of impaired Treg immunosuppressive function.

Recent WGBS analysis comparing primary conventional CD4^+^ T cell and Treg subsets identified an additional 33 Treg-selective demethylated regions (Treg-DRs; Ohkura et al., 2020). Unsurprisingly, several of these Treg-DRs map to transcription factors important for Treg stability, mediators of IL-2 signaling, and immunosuppressive surface molecules, among other genes. We asked whether these Treg-DRs gained methylation in response to repetitive CD3/CD28 stimulation during the manufacturing of the Treg products, which may indicate TCR activation-induced destabilization of the Treg identity. As additional positive and negative controls, we included EPIC array data from FACS-sorted and expanded primary Tregs and CD4mem T cells (*n* = 3). In addition, we had the chance to analyze the final time point of second-generation Treg products, which were generated through GMP-compliant FACS and expanded with the same protocol as the first generation products (*n* = 4; see Methods).

**FIGURE 5:**
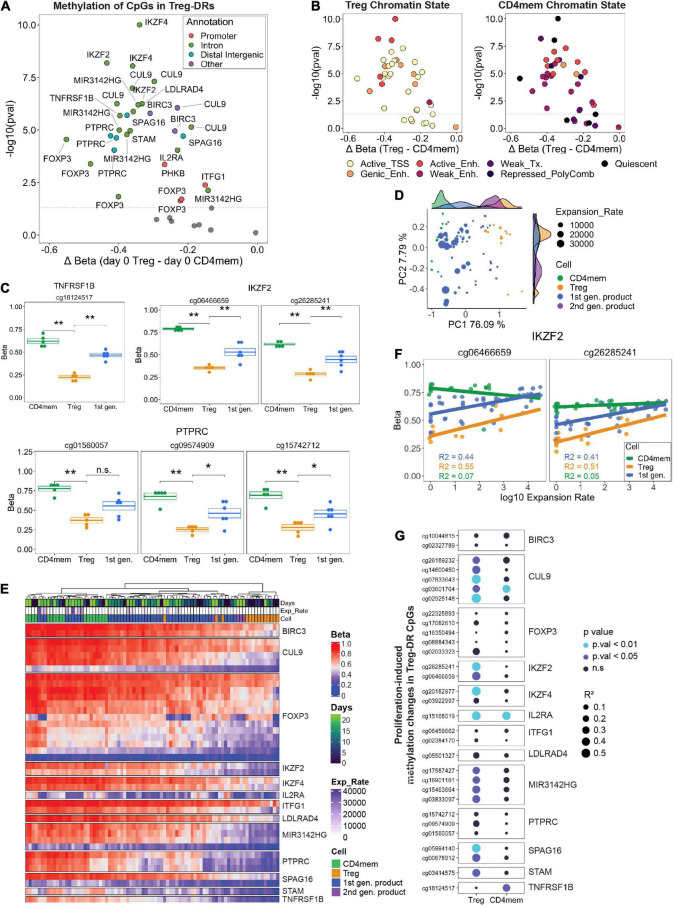
**(A)** FACS-sorted Tregs and CD4 memory T cells (Day 0) were analyzed for CpG methylation at Treg specific demethylated regions (Treg-DRs). Significantly differentially methylated CpGs (*p*. val < 0.05, Student’s *t*-test) were color-coded by genic annotation. **(B)** CpGs in Treg-DRs plotted with the same axes as A, but color-coded by chromatin state annotation (TSS = transcription start site; Enh. = enhancer; Tx = transcription). **(C)** Methylation levels (Beta) of selected Treg-DRs in Treg, CD4mem and Treg products on day 0. *denotes *p*.val < 0.05, **p.val < 0.01, ns = not significant (*T* test). **(D)** PCA analysis of day 0 and day 23 1st generation products, FACS-sorted Tregs and CD4mem as well as day 23 2nd generation products. Histograms at the top and right display representation on PC1 and PC2, respectively, of the sample distribution for each experimental group. **(E)** Unsupervised hierarchical clustering of all samples based on methylation levels of Treg-DRs CpGs. **(F)** DNA methylation change of the *IKZF2* Treg-DR in CD4mem, Tregs and 1st gen Treg product in relation to the expansion rate. *R*^2^ values display the degree of correlation (see **G**). **(G)** Results of linear regression analysis of all differentially methylated Treg-DRs on day 0 (see A) calculating the degree of correlation between gain of methylation and log10 expansion rate. Higher *R*^2^ value (indicated by size of the dot) indicates greater correlation. *P*-values are indicated by color.

We first selected all CpGs from our microarray data which overlapped with the 33 Treg-DRs identified from Ohkura et al. (Supplementary Table 4). As validation, we compared DNA methylation between primary Tregs and CD4mem at day 0, and confirmed that nearly all selected CpGs were significantly hypomethylated in Tregs (Figure 5A), with CpGs mapping to intronic parts of the *FOXP3* gene body displaying the greatest absolute difference. Additionally, these Treg-DR CpGs map to strong, active regulatory regions (e.g., active transcription start sites, genic enhancers that correlate most strongly with gene expression, and additional active enhancers) identified in Tregs (Figure 5B; Roadmap Epigenomics et al., 2015). Strikingly, at the identical genomic location in CD4mem cells, most of these regions were identified to be transcriptionally repressed (e.g., weak enhancer, weak transcription, repressed polycomb region, and quiescent region), further emphasizing that these Treg-DRs are important regulatory features distinguishing Treg identity from that of a CD4mem. We observed that the methylation values of the starting material (day 0) of the first generation products were consistently in between sorted primary CD4mem and Tregs (Figure 5C), confirming that MACS-mediated CD8 depletion followed by CD25 enrichment is not sufficient to obtain a pure Treg starting population.

We were next interested in assessing culture-induced changes in Treg identity and performed a PCA with these selected CpGs to determine how all time points of expanded primary CD4mem T cells, Treg, and first- or second-generation products clustered relative to each other. We found strong separation between CD4mem and Tregs along PC1 (76% of total variation) at any time point, with final first generation products (indicated by highest cumulative expansion rates) clustering more closely with CD4mem cells than final second-generation products (Figure 5D). Indeed, unsupervised hierarchical clustering confirmed that highly expanded final first generation products clustered with the CD4mem cells (Figure 5E), with the selected CpGs showing hypermethylated values relative to primary Tregs. However, primary Tregs also exhibited significantly lower expansion rates, which may partially explain the discrepancy in methylation status of these CpGs.

While the observed similarities between the final first generation Treg products and expanded CD4mem T cells might partially stem from the relative impurity of the MACS-enriched starting material, we found indications that the culture process alone can destabilize the epigenetic identity in primary FACS-sorted Tregs: Although expanded primary Tregs had lower Treg-DR methylation relative to CD4mem T cells at day 23, we were keenly aware that a subset of these selected methylation sites gained methylation with days in culture, approaching similar methylation levels as CD4mem T cells. For example, in both the first generation Treg products and primary Tregs, the selected CpGs within the *IKZF2* (HELIOS) locus showed progressive gain in methylation throughout culture (Figure 5F).

We therefore assessed whether *in vitro* expansion resulted in an epigenetic switch of Treg-DRs in the primary FACS-sorted Tregs. We performed a linear regression on all 31 Treg-DR CpGs that were significantly demethylated in Tregs relative to CD4mem at day 0, and used log10 expansion rates as the dependent variable instead of days in culture to adjust for possible donor-specific differences in day-to-day proliferation behavior. Strikingly, several CpGs mapping to *FOXP3* and *TNFRSF1B* (TNFR2) showed very little change in methylation with increased expansion rate (Figure 5G) indicating a stable demethylated behavior even under strong proliferative conditions. Many of the remaining CpGs, however, displayed significantly higher R^2^ values (> 0.4) which indicates strong correlation between hypermethylation of indicated Treg-DR CpGs and cumulative expansion rate of the population. A similar correlation for many of the same sites could not be observed in the CD4mem controls. This expansion-induced increase in methylation on genes essential for Treg function (e.g., *IKZF2*, *IKZF4*, *IL2RA*), are likely to impact the functional behavior of the Tregs and are of general concern for any Treg product, irrespective of the purity of the starting material.

## Discussion

While adoptive Treg therapy is undoubtedly one of the most promising approaches for intervening excessive inflammatory conditions, standardization of the manufacturing process and quality control remain particularly challenging. As current options for *in vitro* functional testing and phenotype validation are limited, the quality control measures are highly variable between manufacturing sites (Fuchs et al., 2017). In our analyses, we found several lines of evidence that *in vitro* expansion induced by repetitive TCR stimulation leads to progressive remodeling of the DNA methylome, which may impact transcriptional regulation of genes important for Treg function and identity.

Firstly, we observed progressive promoter hypomethylation (activation) of genes known to downregulate T cell activation (e.g., *PDCD1, CD59*, and *IL1RN*) with concomitant promoter hypermethylation (deactivation) of genes that positively regulate TCR signaling (e.g., *RHOH, HLX, RARA*). These methylation changes may indicate that the final Treg product have suboptimal TCR signaling, where an optimally activated state might be more desired.

Secondly, we detected strong promoter hypomethylation in genes implicated in conventional T cell exhaustion, such as *HAVCR2* (TIM-3), *TIGIT*, *LGALS1*, *CD5*, and others, which corresponded to an increase in protein expression on the cell surface. Although these genes have been shown to promote conventional T cell dysfunction during cases of chronic viral infection (Khan et al., 2019), cancer pathogenesis (Scott et al., 2019), and tonic signaling in CAR-T cells (Weber et al., 2021), they also play an active role in enhancing Treg immunosuppressive function (Ukena et al., 2011; Sakuishi et al., 2013; Kurtulus et al., 2015; Sprouse et al., 2018) and hence, might be beneficial for treatment efficacy. This enhanced effector function may come at a cost, as highly suppressive TIM-3^+^ Tregs have impaired survival rates *in vivo*, notably during allograft response during transplant settings (Gupta et al., 2012; Banerjee et al., 2020).

Lastly, we observed progressive hypermethylation of essential Treg-specific demethylated regions (Treg-DRs; Ohkura et al., 2020) during product generation, indicating a destabilization of the Treg identity. Indeed, Tregs have been reported to lose their immunosuppressive phenotype under unfavorable conditions (Marek et al., 2011; Bailey-Bucktrout et al., 2013). In our study, we found significant DNA hypermethylation in a Treg-specific enhancer within the *IKZF2* gene. *IKZF2* encodes for the transcription factor HELIOS, a member of the Ikaros family that has been shown to be a critical regulator of immunosuppressive function. Loss of HELIOS expression has been linked to conversion of Tregs into T effector cells under proinflammatory conditions (Nakagawa et al., 2016). Importantly, the partial remethylation of Treg-DRs also occurred in primary FACS-sorted Tregs and therefore is a concern for improved second-generation Treg products, too, which undergo GMP-compliant FACS sorting for isolation of the starting material.

Although we focused our study on DMRs in transcriptional regulatory elements such as promoters and enhancers, we also observed changes outside regulatory elements, such as in partially methylated domains (PMDs), which largely localize to transcriptionally silent heterochromatic areas (Lister et al., 2011; Hon et al., 2012). While the functional impact of PMD hypomethylation in Tregs remains to be determined, reports have indicated that aberrant PMD hypomethylation may be correlated with cellular senescence and dysfunction (Lister et al., 2011; Schellenberg et al., 2011; Salhab et al., 2018).

Taken together, our study demonstrates that strong *in vitro* expansion leads to epigenetic remodeling at loci important for Treg function and identity during Treg ACT manufacturing, which may ultimately impact long-term persistence or therapeutic efficacy of the product. Whether alternative protocols requiring fewer cycles of TCR-mediated restimulations (MacDonald et al., 2019) or alternative modes of T cell activation (Hippen et al., 2011; He et al., 2017) mitigate the observed epigenetic changes remain to be seen. Prior to our study, characterization of global epigenetic changes throughout Treg or other T cell ACT manufacturing were lacking. However, studies exploiting other *in vitro* and *in vivo* models of frequent T cell activation have reported extensive changes to epigenetic features beyond DNA methylation. For instance, tonic signaling in CAR-T cells results in dynamic genome-wide changes to chromatin accessibility (Gennert et al., 2021), which was similarly found in antigen-specific CD8 T cells responding to chronic viral infections in mice (Sen et al., 2016). Both models demonstrated a marked increase in chromatin accessibility at loci encoding for inhibitory, exhaustion-associated surface markers. Although the effect of long-term frequent stimulation of T cells on histone post-translational modifications is unknown, dynamic changes to both activating and repressive histone modifications can be detected during early activation and differentiation of T cells (Russ et al., 2014; LaMere et al., 2016; LaMere et al., 2017). Due to the clear role of epigenetic regulatory mechanisms on T cell function (and dysfunction), we hope to advocate for the inclusion of epigenetic profiling during manufacturing and quality control assessment of final T cell ACT products, which may uncover opportunities for improving current therapies.

## Data Availability Statement

All DNA methylation data is made available on Gene Expression Omnibus (GEO) Repository (GSE185854).

## Ethics Statement

The studies involving human participants were reviewed and approved by Ethics Committee of the Charité - Universitätsmedizin Berlin. The patients/participants provided their written informed consent to participate in this study.

## Author Contributions

KO performed and coordinated all bioinformatical DNA methylation analyses with contributions from DH and ASa and generated figures with contributions by ASc. KO, ASc, and GG generated DNA methylation profiling data. SS and MS generated and analyzed protein expression data. GZ, LA, DK, and AR generated Treg products under supervision of PR. KO, DH, and JP designed the study, lead the data analysis, and interpretation with contributions by JW, H-DV, MS-H, and PR wrote the manuscript. JP supervised the project. All authors contributed to the article and approved the submitted version.

## Conflict of Interest

The authors declare that the research was conducted in the absence of any commercial or financial relationships that could be construed as a potential conflict of interest.

## Publisher’s Note

All claims expressed in this article are solely those of the authors and do not necessarily represent those of their affiliated organizations, or those of the publisher, the editors and the reviewers. Any product that may be evaluated in this article, or claim that may be made by its manufacturer, is not guaranteed or endorsed by the publisher.

## Footnotes

1
https://github.com/steschlick/nautilus

2
https://cran.r-project.org/web/packages/ggpubr/index.html

3
http://corybrunson.github.io/ggalluvial/

4
https://cran.r-project.org/web/packages/ggExtra/index.html
