# Supplementary figures and images for: Strong Expansion of Human Regulatory T Cells for Adoptive Cell Therapy Results in Epigenetic Changes Which May Impact Their Survival and Function
Source: Front Cell Dev Biol. 2021 Nov 18;9:751590. doi: 10.3389/fcell.2021.751590 (PMC8639223; doi:10.3389/fcell.2021.751590)

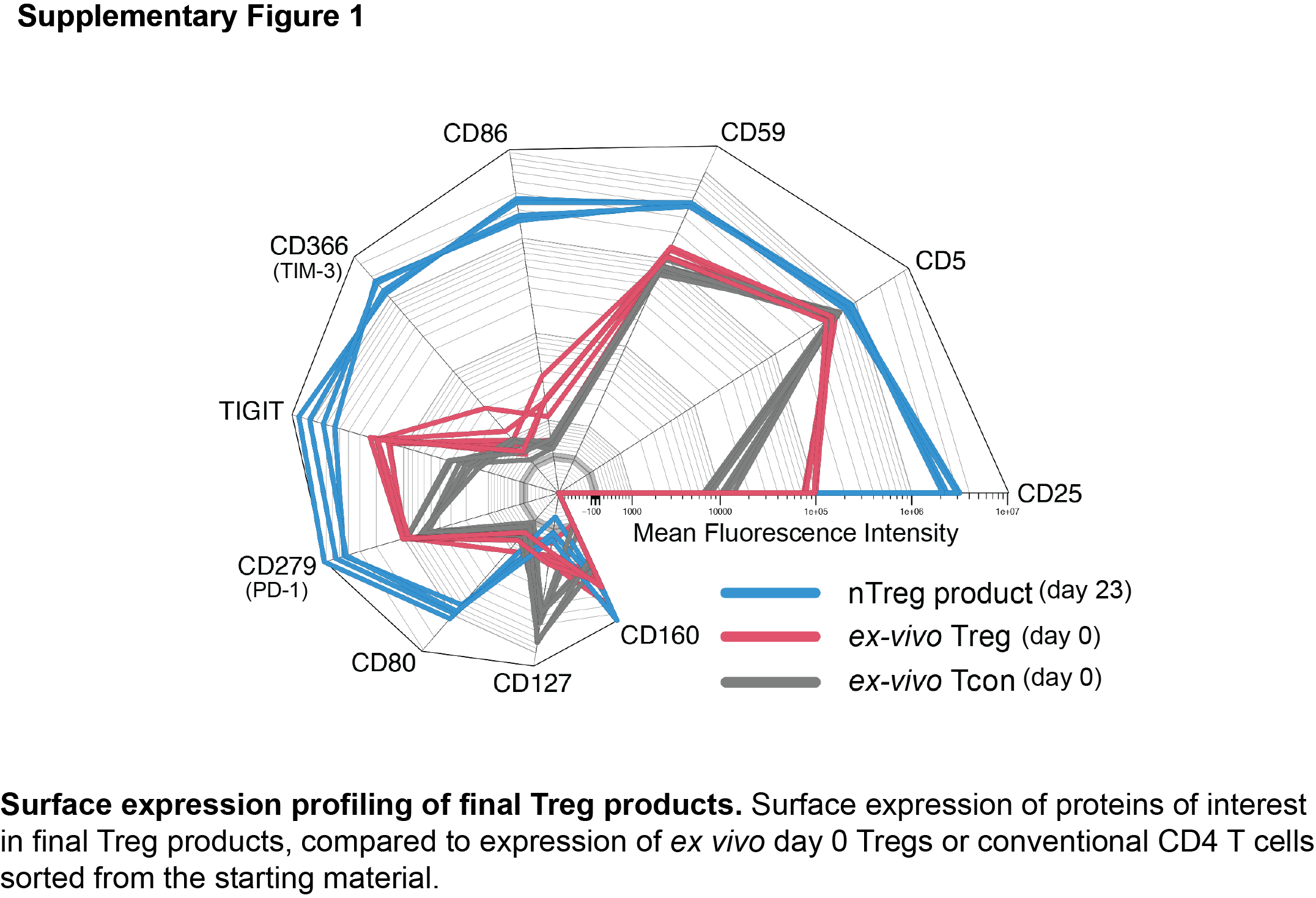

Supplement: Supplementary file 5 [file Image_1.png]
